# Evaluating diameter distribution series of small-leaved lime (*Tilia cordata* Mill.) in forest stands

**DOI:** 10.1186/s13007-021-00741-6

**Published:** 2021-04-26

**Authors:** Aydar Gabdelkhakov, Zagir Rakhmatullin, Maria Martynova, Ilyas Fazlutdinov, Ilnur Mullagaleev

**Affiliations:** Department of Forestry and Landscape Design, Bashkir State Agrarian University, Ufa, 450001 Russian Federation

**Keywords:** Small-leaved lime, Forest plantations, Planting density, Average diameter, Statistical indicators, Pearson curves, Weibull function

## Abstract

**Background:**

The paper provides studies on the structure of planted small-leaved lime (*Tilia cordata* Mill.) in the conditions of the Bashkir Cis-Urals. This work aimed to analyze their assortment by diameters and compile appropriate tables. This is the first study of lime for this region. The results of the study are based on data from 69 temporary sample plots. Stands are represented by trees of 11–79 years old, not affected by thinning. They belong to the I–III growth classes and the most common goutweed forests.

**Results:**

It was found, that small-leaved limes have a specific structure—coefficient of diameter variation in stands decreases with a higher average diameter, reaching 26 and 16% at 28 cm with thin and dense initial planting, respectively. The variability of tree diameters is related to the average stand diameter and is due to the initial density of the grown plantations. Correlations between the coefficients of asymmetry and excess with age and the average diameter was revealed.

**Conclusions:**

The verification of theoretically calculated frequencies of distribution series to empirically observed frequencies showed a discrepancy in 29 and 19% of the total number, respectively. For the remaining series of distributions, the scale and shape parameters of the Weibull function were modeled using the average, minimum and maximum diameters, standard deviation, coefficients of asymmetry and excess. This made it possible to develop stand tables for small-leaved limes depending on the average diameter.

## Introduction

The application of forest stand structure and development regularities is an integral part of forest management planning, decision-making, scientific research. It leads to higher forest quality and productivity, determines economic efficiency. The structure of stands affects the intensity of biogeocenotic processes, the efficiency of production and deposition of organic matter, the stability and biosphere functions of forest ecosystems. The stand structure is understood as a cumulative combination of examination indicators variation, the distribution of trees by these values, their relationship at certain age stages of the forest community development.

When studying the structure of stands, the analysis of trunk diameter variation is of the greatest interest. The diameter at breast height (d_1,3_) is an important indicator of a tree. It is widely used in forest inventory to calculate some stand parameters such as stem volume and aboveground biomass that cannot be measured directly. The d_1,3_ distribution is an indicator of the forest structure [1], its stock, assortment composition, and so on [2–4].

There is continuing interest in studying the structure of stands, despite more than a century of history [5–7]. The stand structure is analyzed in terms of different tree species [1, 8, 9], composition [10–12], average age [4, 13], age structure [10, 14], growth site conditions [3, 15], density [4], factors that affect the tree number distribution by diameter class [3, 6, 16]. Bassil et al. [17] studied the temporal stability of the number of trees by diameter classes in different-aged Northern hardwoods that are subject to logging [17]. Many publications are aimed at searching for models and evaluating their effectiveness in describing the d_1,3_ distribution [1, 3, 18], including those subjected to thinning [13]. Thus, Safonov et al. [12] describe the functionality of the Forest-Fit software package and its features that simplify the estimation of common probability distributions for modeling d_1,3_ distributions, using the example analysis of mixed *Pinus ponderosa* forest compartments. Paradis and Lebel [19] propose a methodology that can be used to build a d_1,3_ distribution model for any combination of forest species and types in Quebec (Canada), using easily accessible data from the state program for the inventory of sample areas.

Though there is a significant amount of literature on distribution series of different tree species and natural conditions, studies on small-leaved limes (*Tilia cordata* Mill.) are not numerous [10, 15, 20].

Much attention is paid to determining the structure of planted stands [16, 21, 22], because even the same species, due to differences in the initial density, tree distribution, tree ages, the quality of planting material and different growing conditions, will form a variety of structures.

Pure and mixed artificial stands of small-leaved limes have been planted in the Bashkir Cis-Urals since the end of the 30 s of the last century. There have been no studies describing their structure and dynamics. Since forest stand structure regularities by estimation indicators are statistical in nature, there is a need to develop differentiated stand inventory standards [2]. Therefore, the goal of the work is to consider diameter distribution characteristics in planted stands of small-leaved lime and to compile appropriate tables for the conditions of the Bashkir Cis-Urals.

## Materials and methods

The paper studies the diameter structure of 11–79-year-old small-leaved lime plantations in 69 temporary sample plots located in the Bashkir Cis-Urals and belonging to the forest-steppe zone of the South Ural forest-steppe region of the European part of the Russian Federation. According to the S.F. Kurnaev’s fractional forest zoning, this region belongs to the broad-leaved forest zone of the forest-steppe subzone within the Russian plain [23].

**Fig. 1:**
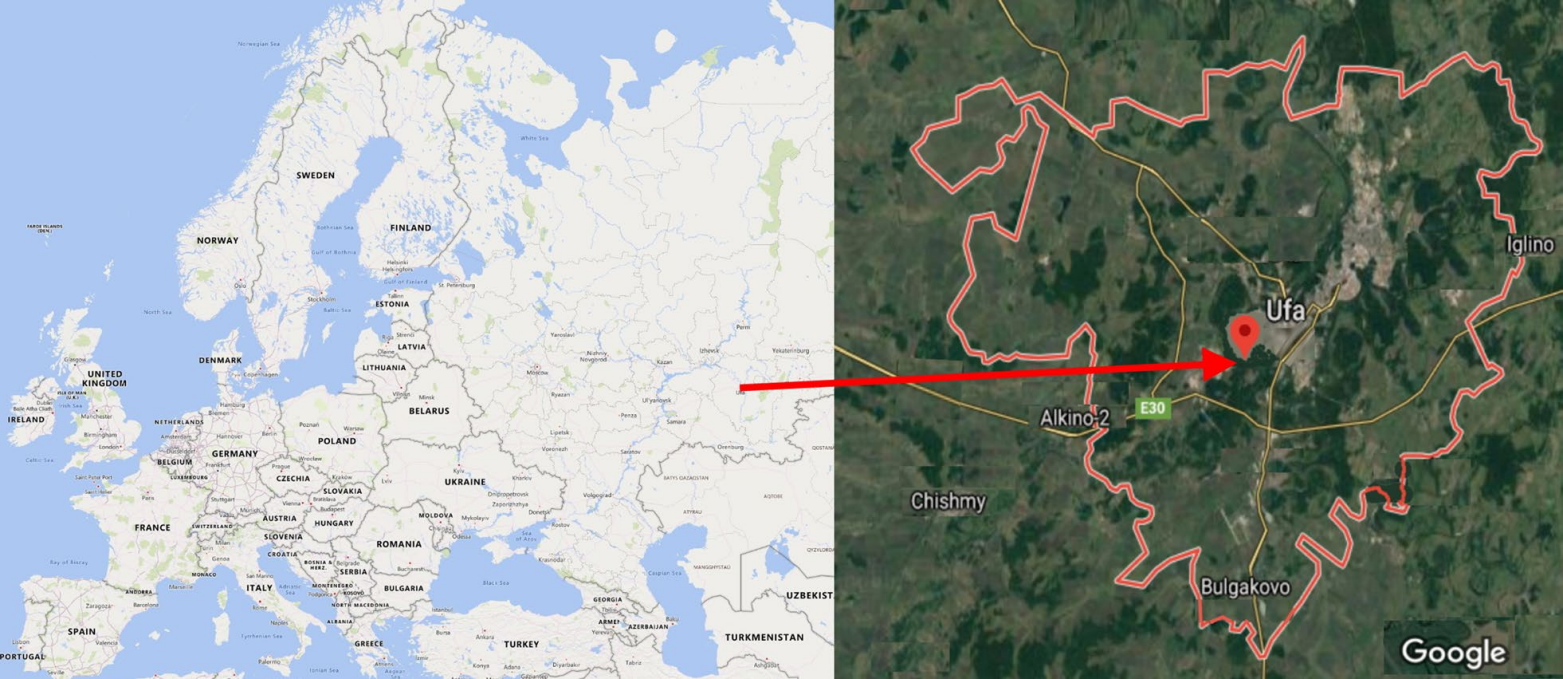
Spatial location of the data collection area

The studied plots (except for three sample areas) are located on the territory of Ufa city and the Ufa municipal district, located at 54°70′ north longitude and 55°90′ east longitude 150 m above sea level (Fig. 1). The climate of the district is continental, rather humid. The average annual air temperature is 3.0 °C, the average temperature in January is − 14.5 °C, in July 19.5 °C with an absolute maximum of 40 °C and an absolute minimum of − 50 °C. The average annual precipitation is within 500–600 mm. It is about 350 mm during the growing season [24]. In these conditions, small-leaved limes develop according to the I–III growth classes and represent productive phytocenoses.

The data was collected from rectangular sample plots of 0.1 ha or more, depending on the stand density. Each plot is a homogeneous plantation. All trees > 3.9 cm were d_1,3_ estimated by one-centimeter diameter class in every plot. Tree heights were measured. The age is determined based on the data of mensurational descriptions and the list of forest plantations. The remaining stand estimation indicators were calculated based on the trees counted [2].

The studied plantations were of different densities, and were not thinned. They are grouped for an analysis as follows: thin—from 2 to 6 thousand pieces per ha (52 plots), medium density—from 6 to 11 thousand pieces per ha (10 plots), dense and very dense—from 11 to 20 thousand pieces per ha (7 plots). The main dendrometric characteristics of the selected plantings are shown in Table 1.

**Table 1:** Main dendrometric characteristics of the selected sites

Parameter	Average	Minimum	Maximum	Coefficient of variation, %
Thin density (n = 52)				
Age, years	50	11	79	31
The number of trunks, pieces/ha	1472	407	4024	67
Average height, m	17	3	26	22
Average diameter, cm	17	3	26	29
Medium density (n = 10)				
Age, years	46	32	60	20
The number of trunks, pieces/ha	2786	1366	4824	47
Average height, m	16	14	18	7
Average diameter, cm	14	11	17	16
Thick and very thick (n = 7)				
Age, years	47	26	67	26
The number of trunks, pieces/ha	2504	1586	4850	43
Average height, m	16	10	20	19
Average diameter, cm	14	8	19	26

Basic statistics are calculated for all series of d_1,3_ distributions: sample size (n), arithmetic mean (X), standard deviation (S), coefficients of variation (C_v_), asymmetry (A_s_), excess (E_x_), and others. By conducting a correlation analysis, the relationships between evaluation indicators and statistics of the d_1,3_ distribution series of trees were revealed, being approximated by the following functions:

1 $$y_{i} = b_{0} \cdot D^{{b_{1} }}$$

2 $$S = b_{0 } + b_{1} \cdot d_{min} + b_{2} \cdot d_{max} + b_{3} \cdot D$$

3 $$A_{s} \left( {E_{x} } \right) = b_{0} + b_{1} \cdot D + b_{2} \cdot d_{min} + b_{3} \cdot d_{max} + b_{4} \cdot S$$

where y_*i*_ is the minimum (*d*_*min*_) and maximum (*d*_*max*_), arithmetic mean (X) values of the diameters of the distribution series (in cm); b_*i*_ is the coefficients of models; D is the average inventory diameter of the stand, cm.

Tree diameter distribution series statistics (X, d_min_ and d_max_, S, A_s_ and E_x_) was compared by calculating the mean-square deviation of pairwise matched models (σ, %) [25]:

4 $$\sigma = 200\sqrt {\frac{{\mathop \sum \nolimits_{i = 1}^{n} \left( {\frac{{a_{i} - c_{i} }}{{a_{i} + c_{i} }}} \right)^{2} }}{n - 1 }}$$

where a_*i*_ and C_*i*_ are pairwise compared data of distribution statistics calculated from models 2–4; n is the number of pairs compared, pcs.

To describe these distribution series, the Pearson family curves of I–VII types were used [10]. The number of trunks was equalized by one-centimeter diameter classes in Microsoft Excel. The correspondence of theoretically calculated frequencies of distribution series to empirically observed frequencies was estimated by the B. S. Iastremskii criterion [26].

Since there was no special software for leveling the curves of the Pearson family, a two-parameter Weibull distribution in the Statistica environment was used to calculate the expected values of d_1,3_ frequencies with an accuracy of up to a centimeter, expressed as a percentage of the total number of trunks of each sample plot. It has the following density function (for positive parameters γ and β):

5 $$f\left( x \right) = \left( {\begin{array}{*{20}c} {\begin{array}{*{20}c} \gamma \\ - \\ \end{array} } \\ \beta \\ \end{array} } \right)\left( {\begin{array}{*{20}c} {\begin{array}{*{20}c} x \\ - \\ \end{array} } \\ \beta \\ \end{array} } \right)^{{\left( {\gamma - 1} \right)}} e^{{\left[ { - \left. {\left( {\begin{array}{*{20}c} {\begin{array}{*{20}c} x \\ - \\ \end{array} } \\ \beta \\ \end{array} } \right)^{\gamma } } \right]} \right.}}$$

where γ is the parameter of the distribution form; β is the distribution scale parameter; *e* is the Euler number (2.71…).

Estimation accuracy of the Weibull function was verified using the Kolmogorov–Smirnov test (K–S) and the Anderson–Darlig test (AD) with a probability of 5%. After evaluating the γ and β parameters of this function, a correlation and regression analysis was performed and multiple regression models were obtained to predict them depending on the distribution statistics (X, d_min_ and d_max_, S, A_s_ and E_x_).

## Results

The tree diameter is the main examination indicator for studying the structure of the stand. It varies from 1 to 47 cm by sample plots. The average arithmetic value of d_1,3_ for individual objects ranges from 2.6 cm to 25.4 cm. The coefficient of variation, which is an indicator of the homogeneous structure of the stand, varies from 5 to 56%. In three sample plots, the d_1,3_ distribution is characterized by left—sided asymmetry (from − 0.23 to − 0.01), while it is right-sided in the rest. it is insignificant in five plots (from 0.01 to 0.09). The coefficient of asymmetry in other compartments ranges from 0.11 to 1.93. D_1,3_ distributions are characterized by different density grades from − 0.97 to 5.61. Analysis of the asymmetry and excess coefficients for 34 and 13 sample plots, respectively, showed that they are beyond their twofold basic errors. This indicates the difference between the given series from the standard one. For the rest of the sample areas, the asymmetry and excess confidence estimation by the t-Student’s test, on the contrary, shows that there is no deviation of the distribution curves from the standard one (t_0, 05_ < 1.97).

To find out the regularities of the diameter distribution dynamics and its statistics, a correlation analysis was performed (Table 2).

**Table 2:** Correlation coefficients of distribution series

Indicator	S	d_*min*_	d_*max*_	C_v_	A_s_	E_x_
Thin density (n = 52)						
A	*0.713*	*0.491*	*0.814*	− *0.358*	− 0.032	0.029
D	*0.714*	*0.759*	*0.855*	− *0.570*	− *0.312*	− 0.232
X	− 0.255	− *0.459*	− *0.361*	*0.306*	0.244	− 0.243
g	*0.670*	*0.787*	*0.831*	− *0.616*	− *0.345*	0.156
R_d_	− 0.003	*0.717*	0.269	− *0.962*	− *0.624*	− *0.429*
Medium density (n = 10)						
A	− 0.256	− 0.457	− 0.352	− 0.211	− 0.288	*0.782*
D	− *0.664*	− *0.844*	− 0.511	0.078	− *0.675*	0.408
X	− 0.418	0.079	− *0.787*	− *0.760*	− 0.013	0.027
g	0.547	0.446	*0.969*	0.583	0.133	0.040
R_d_	0.394	*0.765*	0.520	− *0.981*	0.075	− 0.141
Thick and very thick (n = 7)						
A	0.710	*0.864*	*0.826*	− *0.877*	− 0.050	0.018
D	*0.882*	*0.980*	*0.913*	− *0.855*	0.001	− 0.093
X	− 0.410	− 0.417	− 0.489	0.015	− 0.440	− 0.247
g	*0.870*	*0.980*	*0.909*	− *0.866*	0.011	− 0.085
R_d_	0.394	*0.765*	0.520	− *0.981*	0.075	− 0.141

A: forest plantation age, years; g: forest plantation density, trees/ha; R_d_: rank of the average tree by diameter; the italic values of correlation coefficients are significant at *p* < 0.05

The studied limes showed the following changes in the distribution series with tree aging. The relation of the asymmetry and excess coefficients with age is not expressed, but there is a tendency to decrease the asymmetry coefficient and increase the excess coefficient with increasing age. The coefficient of variation significantly decreases with the age of plantations. The standard deviation increases with aging and higher average diameters of thin and dense plantations. The value of the average tree rank for thin planting significantly correlates with the coefficient of asymmetry (r = − 0.624) and the minimum diameter of the tree in the distribution series (r = 0.717). The identified relationships made it possible to create models (1–3), presented in Table 3.

**Table 3:** Parameter estimates and accuracy of fitting models (1–3) describing dependencies of distribution series statistics

Indicator*	b_0_	b_1_	b_2_	b_3_	b_4_	F	R^2^	S_e_	S_m_
Thin density (n = 52)									
*d*_*min*_	0.27088	1.11063	–	–	–	87	64	0.321	0.244
*d*_*max*_	3.42064	0.78163	–	–	–	220	81	0.142	0.113
*X*	0.83024	1.04935	–	–	–	17,865	99	0.021	0.016
S	0.66974	− 0.28426	0.07697	0.21574	–	86	84	0.569	0.373
A_s_	0.12061	− 0.19619	0.13502	0.05859	0.18010	14	59	0.198	0.142
E_x_	− 0.74550	− 0.23978	0.12409	0.17177	− 0.29138	26	73	0.279	0.203
Medium density (n = 10)									
*d*_*min*_	0.08156	1.59305	–	–	–	13	61	0.223	0.166
*d*_*max*_	1.43776	1.09182	–	–	–	37	82	0.092	0.073
*X*	0.87370	1.03663	–	–	–	1760	99	0.012	0.010
S	0.96089	− 0.00135	0.13595	− 0.03836	–	7	79	0.386	0.242
A_s_	0.81713	− 0.14320	0.14306	0.09346	− 0.41614	9	87	0.134	0.083
E_x_	− 0.38934	− 0.02754	0.07582	0.16829	− 1.02149	24	95	0.134	0.079
Thick and very thick (n = 7)									
*d*_*min*_	0.04068	1.88426	–	–	–	123	96	0.119	0.083
*d*_*max*_	3.22645	0.77014	–	–	–	19	79	0.123	0.100
*X*	0.89798	1.02827	–	–	–	9146	99	0.007	0.005
S	0.67516	− 0.17861	0.03643	0.21802	–	5	82	0.361	0.203
A_s_	0.79212	− 0.04337	0.04025	0.03288	− 0.26396	*1*	66	0.134	0.061
E_x_	− 0.11756	0.11425	− 0.21710	0.08867	− 0.69883	*3*	85	0.173	0.084

Where *F* is the significance of the equation; *R*^2^ is the coefficient of determination, %; *S*_e_ is the standard equation error; *S*_m_ is the average absolute error of the equation; the italic values of F-criteria is not relevant (*p* > 0.05)

The data in Table 3 indicate the significance of the obtained equations (except for two cases) and the possibility of their application for forecasting.

**Fig. 2:**
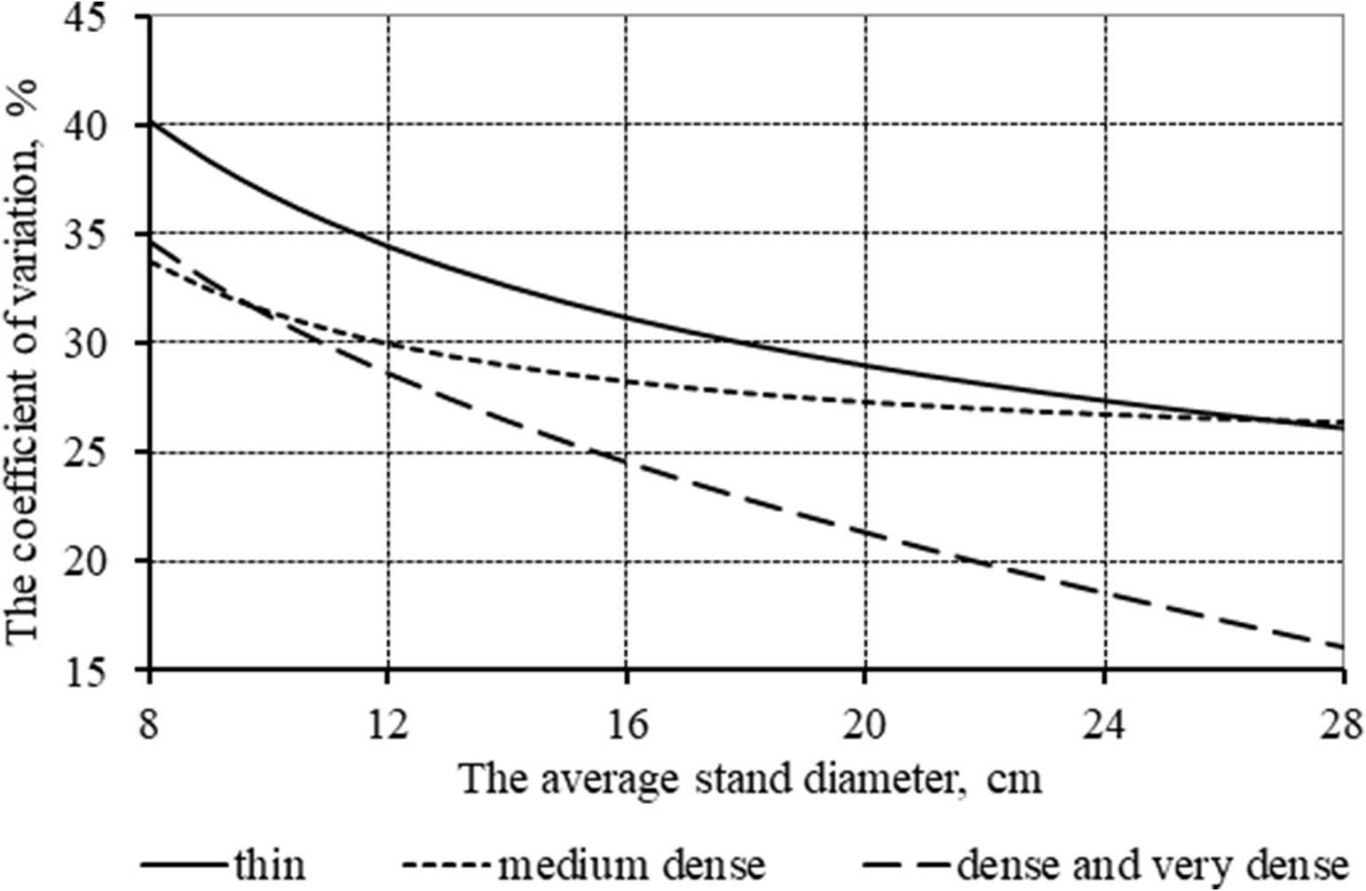
Aligned indicators of tree diameter variability in stands of different planting density

Checking the statistics of the distribution series according to formula 4 using the developed models (1–3, Table 3) showed that the initial density of plantations (within the considered planting schemes) affects them: the degree of difference is significant—for *d*_*min*_ (27–101%), *d*_*max*_ (16–19%) and S (18–21%). A similar test for the coefficients of asymmetry and excess demonstrated several times greater differences: up to 535% for A_s_, up to 1817% for E_x_. Differences in the distribution series depending on the density of forest plantation can be visually estimated from Fig. 2, which illustrates aligned indicators of variability in tree diameters.

The given data indicate that diameters in thin plantations vary greatly compared to dense and very dense stands. Meanwhile, medium dense plantations have an intermediate position of variability. With average diameters of 8–14 cm, the variability is closer to dense and very dense stands, and with large diameters (22–28 cm)—to thin. With higher average stand diameter, the variability of trees in thickness decreases, reaching values of 26 and 16% at 28 cm with thin and dense initial planting, respectively.

**Fig. 3:**
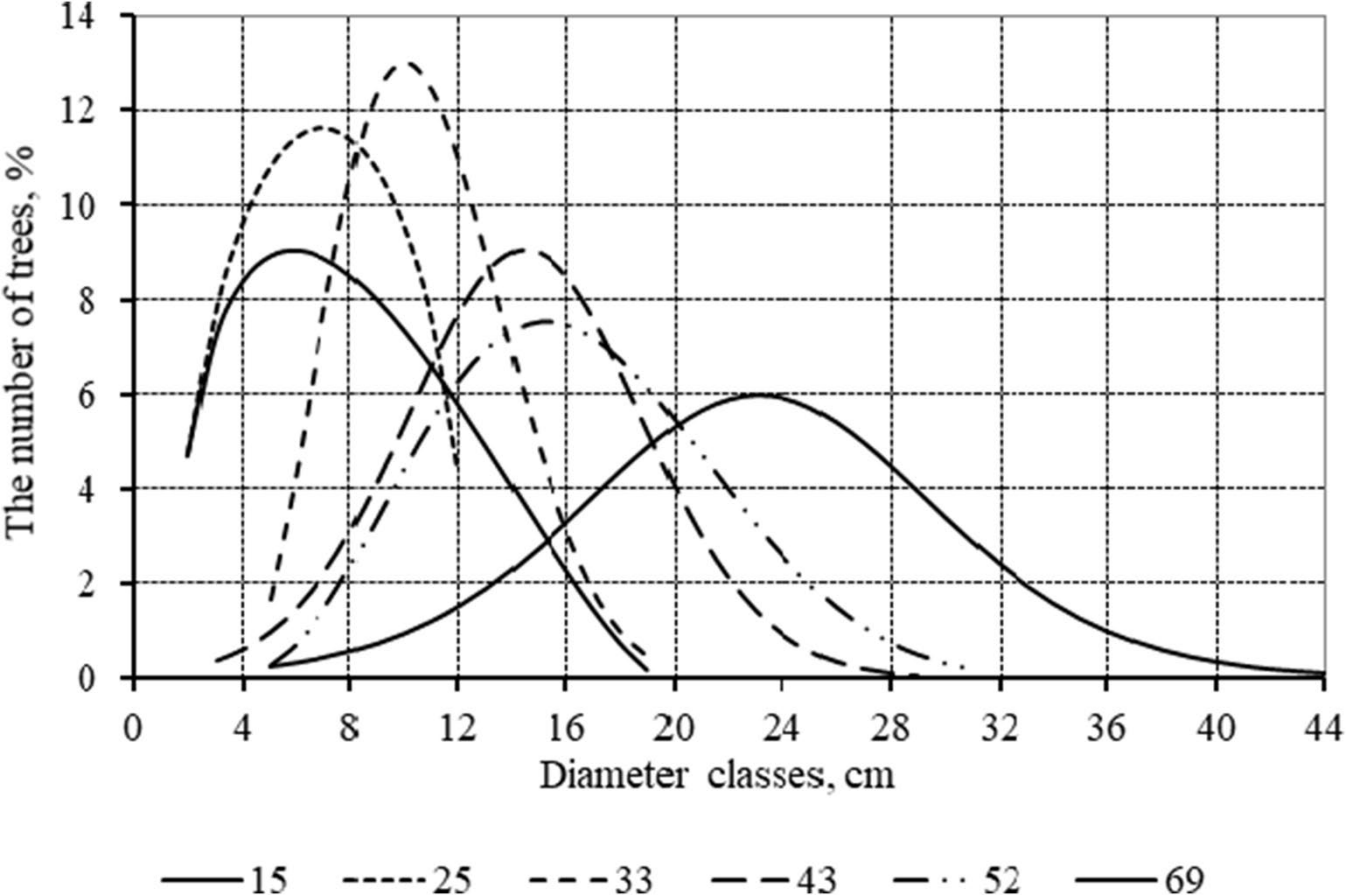
Aligned indicators of diameter distribution in stands aged

The use of Pearson curves to approximate the tree distribution series by diameter showed that the type I function provides more satisfactory description for 46 of the 69 sample plots, types II and VII for 7 plots, type IV for 6 sample compartments, type VI for the rest. This indicates the heterogeneity of the tree distribution by their size with the age of stands. The verification of theoretically calculated frequencies of distribution series to empirically observed frequencies by the B.S. Iastremskii criterion proved to be relevant for 49 applied models (71%). Figure 3 presents the simulation results for six sample plots with the same initial planting density (thin, row spacing 3.0 m, plant spacing 0.7 m).

It shows that trees of the smaller diameter classes are more frequent in young stands and their number decreases with age. At the same time, as the forest grows, the frequency of large diameter trees increases, shifting the curve to the right. With a higher average diameter of the stand, the distribution curves stretch, they become less steep and less elongated upwards in the center, approach the abscissas axis more slowly when moving away from the middle, and become the closest to the standard distribution.

Due to the small number of samples of medium, dense and very dense plantations, insignificant correlations between the coefficients of asymmetry and excess with the average diameter of stands (Table 2), as well as the deviation of two models (Table 3), the following calculations were performed only for stands with thin planting density. The distribution series by one-centimeter diameter classes as a percentage were estimated by the two-parameter Weibull function. The model validity test evaluated by the K–S and AD criteria proved their adequacy for 42 sample plots, since the values of these tests were not significant (p > 0.05). This condition indicates that the observed and expected frequencies are not statistically different from each other, and therefore the null hypothesis cannot be rejected. On the other hand, for 10 sample areas (19%), significant values of the K–S and AD tests indicate inadequate estimates of diameter frequencies (p < 0.05).

A preliminary linear correlation analysis was performed to assess trends between the two parameters of the Weibull function and the distribution statistics (X, d_min_, d_max_, S, A_s_, and E_x_) (Table 4).

**Table 4:** Correlation coefficients of distribution series

Indicator	X	d_*min*_	d_*max*_	S	A_s_	E_x_
β	*0.9988*	*0.8234*	*0.9182*	*0.6586*	− 0.0869	*0.3732*
γ	*0.5318*	*0.7639*	0.1599	− 0.3018	− *0.5457*	0.0390

The italic values of correlation coefficients are significant at *p* < 0.05

The data in Table 4 indicates a high chance of successfully projecting the probability distribution by restoring the coefficients of the Weibull function by multiple regression. The following models were offered:

6 $$\begin{aligned} \beta & = - 0.0131274 + 1.04403*X - 0.0162531*d_{min} + 0.00710392*d_{max} \\ & \quad + 0.190799*S + 0.0188951*As - 0.041876*Ex \\ F & = 59364; R^{2} = 99\% ; S_{e} = 0.051; S_{m} = 0.032 \\ \end{aligned}$$

7 $$\begin{aligned} \gamma & = 3.88558 + 0.210968*X + 0.12539*d_{min} - 0.0998994*d_{max} \\ & \quad - 0.249702*S - 0.640852*As + 0.572217*Ex \\ F & = 139; R^{2} = 96\% ; S_{e} = 0.192; S_{m} - 0.129 \\ \end{aligned}$$

Both models presented have high F scores (p < 0.0001) and explain 99 and 96% of the variability, respectively.

The coefficients obtained for the Weibull function were used to estimate the evolution of the diameter distribution in planted stands of small-leaved lime depending on the class of the average diameter (one-centimeter diameter class), the calculated values of which are given in Appendix.

## Discussion

Planted stands of small-leaved limes in distribution series by d_1,3_, being the main forest inventory indicator, change the place of the average tree, the reduction numbers by rank, the minimum and maximum diameters, the concentration of the trunk number percentage in the diameter class, the standard deviation value, asymmetry and excess of the series, which is consistent with other studies [2, 4, 6].

The use of Pearson curves made it possible to effectively describe the d_1,3_ distribution series. This is consistent with the view of Bachioua [27], who considers that they can flexibly adapt to a set of well-known theoretical and practical distributions. The work of Shakil et al. [28] showed a better result of the proposed Pearson model than the gamma, lognormal and inverse Gaussian distributions.

The discrepancy between the theoretically calculated frequencies of the distribution series and the empirically observed frequencies of the Pearson curves and the two-parameter Weibull function (29 and 19% of the total number, respectively) indicates the complex nonlinear nature of the d_1,3_ distributions and modeling the probability-density function, which conforms to the research of Diamantopoulou et al. [29] and Pach and Podlaski [1].

Many authors report that the Weibull function is widely used in many forestry applications to model d_1,3_ distributions [3, 13, 22], because it has a simple cumulative function and is flexible in selecting distributions for different forms and degrees of asymmetry [11, 29, 30]. Moreover, the simple two-parameter form of its distribution often gives better results compared to the three-parameter form [3, 6, 30]. In addition, the two-parameter Weibull function was used in a large number of related studies due to the high correlation of its parameters (γ and β) with the stand characteristics [13, 17, 18]. Therefore, its application is suitable for describing the d_1,3_ distributions in this study.

The correlation analysis results obtained between the two parameters (γ and β) of the Weibull function and the characteristics of *d*_*min*_ and *d*_*max*_ are similar to those observed by Binoti et al. [13]. Except that there is no connection between *d*_*max*_ and γ. Linear models that link the parameters β and γ to the main distribution statistics show very high determination coefficients (99 and 96%, respectively). It is noted that their performance deteriorates as the average value of d_1,3_, asymmetry and excess of the distribution series increases, which is consistent with studies by Lima et al. [3].

Diameter distribution models can be useful in describing and analyzing the stand structure, age distribution, growth and yields. They can be employed in assessing the stand stability and calculating a number of trees in each diameter class to plan forest management activities, reveal previous violations, predict successions of forests and land biomass reserves, etc. [3, 4, 12]. Tree diameter distribution modelling based on remote sensing by laser scanning also requires indirect estimation of these distributions using forecasting models [22]. Determining stand diameter distribution is costly due to measuring diameters for a large number of trees during inventory. Costs can be reduced by using diameter distribution models based on diameter classes, depending on the average diameter, the number of trunks and the stand basal area [22, 30]. However, the given models should be treated with caution, since these stand parameters are used in many different cutting strategies resulting in different diameter distributions [6]. It becomes particularly evident for forest crops of different densities, especially during the stand formation, since there is a different number of trunks and the stand basal area at the same average diameter. In practical application, such models, being a gross simplification of the reality, can be disaggregated into more detailed resolutions, providing the forest manager with more detailed information [6].

## Conclusions

The given paper analyzes the diameter distribution of small-leaved limes in forest plantations. Lime plantations were found to have certain features in the structure due to the even age, planting density and biological characteristics as a shade-tolerant specie. There is regularity in variation coefficient changes. It significantly decreases with the age of plantations. The experimental material revealed correlations between the coefficients of asymmetry and excess with the age and average diameter of small-leaved lime.

The analysis of the numerical characteristics and models of the structure of plantings by diameter gives grounds to consider the initial density of the grown plantations as the determining factor.

The analysis of tree distribution by diameter classes using the Pearson curves and the Weibull function provided the best processing of the experimental material. The simplicity of algebraic manipulation and the ability to take various forms of distribution curves make Pearson curves and the Weibull function a useful tool for forestry models.

In fact, this is the first study of this type for planted stands of small-leaved lime. The obtained data of the compiled distribution series of the total number of trunks by diameter class are recommended for describing and analyzing the structure of stands, for forest management (conducting various logging operations), development of forest inventory standards (drawing up commodity tables, guidelines for thinning), assessing sustainability, environmental factors, inventory, etc.

## Data Availability

All data will be available on request.

## Appendix

See Table 5.

**Table 5:** Diameter distribution depending on the average diameter of small-leaved lime trees in the Bashkir Cis-Urals

Diameter class, cm	Number of trees for an average stand diameter (cm), %									
	8	9	10	11	12	13	14	15	16	17
2	2.0	1.5								
3	4.4	3.5	2.0	1.4	1.1					
4	7.9	5.8	4.0	2.9	2.1	1.5	1.0			
5	11.3	8.0	6.0	4.5	3.4	2.5	1.9	1.3		
6	13.4	10.0	8.0	6.2	4.8	3.6	2.8	2.2	1.0	1.0
7	13.8	11.9	9.9	7.8	6.2	5.0	3.8	3.1	1.6	1.7
8	13.2	12.5	10.9	9.3	7.6	6.2	4.9	4.1	2.4	2.4
9	11.1	11.8	11.2	10.3	8.7	7.4	6.0	5.0	3.2	3.2
10	8.8	10.4	10.9	10.5	9.3	8.2	7.0	5.9	4.0	4.0
11	6.3	8.6	10.1	10.1	9.6	8.8	7.8	6.7	4.8	4.9
12	4.0	6.3	8.3	9.2	9.4	9.1	8.2	7.4	5.7	5.7
13	2.0	4.4	6.5	7.9	8.8	8.9	8.4	7.9	6.4	6.4
14	1.0	2.8	4.6	6.4	7.7	8.4	8.3	8.2	7.1	6.9
15	0.5	1.3	3.1	4.9	6.4	7.5	7.8	8.1	7.6	7.3
16	0.2	0.6	1.9	3.4	5.0	6.2	7.1	7.7	7.8	7.4
17	0.1	0.3	1.2	2.3	3.6	5.0	6.2	7.0	7.7	7.3
18		0.2	0.7	1.4	2.5	3.9	5.2	6.1	7.4	7.0
19		0.1	0.4	0.8	1.6	2.9	4.1	5.1	6.8	6.5
20			0.2	0.4	1.0	2.0	3.1	4.2	6.1	5.9
21			0.1	0.2	0.6	1.3	2.3	3.3	5.1	5.2
22				0.1	0.3	0.8	1.6	2.4	4.2	4.4
23					0.2	0.5	1.1	1.7	3.4	3.6
24					0.1	0.2	0.7	1.2	2.6	2.8
25						0.1	0.4	0.7	1.9	2.1
26							0.2	0.4	1.3	1.5
27							0.1	0.2	0.9	1.1
28								0.1	0.6	0.8
29									0.3	0.5
30									0.1	0.3
31										0.1
Total	100.0	100.0	100.0	100.0	100.0	100.0	100.0	100.0	100.0	100.0

Diameter class, cm	Number of trees for an average stand diameter (cm), %								
	18	19	20	21	22	23	24	25	26
6	0.5								
7	1.1	1.0	0.6						
8	1.8	1.6	1.1	0.9	0.8				
9	2.5	2.2	1.6	1.3	1.0	0.6	0.5	0.3	
10	3.2	2.8	2.2	1.8	1.4	1.0	0.8	0.5	0.5
11	4.0	3.5	2.8	2.3	1.8	1.4	1.1	0.8	0.7
12	4.7	4.1	3.4	2.8	2.3	1.9	1.5	1.1	1.0
13	5.4	4.7	4.0	3.4	2.8	2.4	1.9	1.5	1.3
14	6.0	5.3	4.6	3.9	3.3	2.9	2.3	1.9	1.7
15	6.6	5.9	5.2	4.5	3.8	3.4	2.8	2.4	2.0
16	7.0	6.4	5.7	5.0	4.4	3.9	3.3	2.8	2.4
17	7.2	6.7	6.1	5.6	4.9	4.5	3.8	3.3	2.8
18	7.1	6.9	6.5	6.1	5.4	5.0	4.2	3.7	3.3
19	6.8	6.9	6.7	6.4	5.8	5.4	4.7	4.2	3.7
20	6.4	6.7	6.7	6.6	6.1	5.8	5.1	4.6	4.1
21	5.8	6.3	6.5	6.6	6.4	6.0	5.5	5.0	4.5
22	5.2	5.7	6.2	6.4	6.4	6.2	5.8	5.4	4.9
23	4.5	5.1	5.7	6.0	6.3	6.2	5.9	5.7	5.2
24	3.7	4.4	5.1	5.5	6.0	6.1	6.0	5.8	5.5
25	3.0	3.6	4.5	5.0	5.6	5.9	5.9	5.9	5.7
26	2.3	2.9	3.8	4.4	5.1	5.5	5.7	5.8	5.8
27	1.7	2.2	3.0	3.8	4.5	5.0	5.4	5.6	5.7
28	1.3	1.7	2.4	3.1	3.8	4.4	5.0	5.3	5.4
29	0.9	1.2	1.8	2.5	3.1	3.8	4.5	4.9	5.1
30	0.6	0.9	1.3	1.9	2.5	3.2	4.0	4.4	4.8
31	0.4	0.6	0.9	1.4	2.0	2.5	3.4	3.9	4.4
32	0.2	0.4	0.6	1.0	1.5	2.0	2.9	3.4	4.0
33	0.1	0.2	0.4	0.7	1.1	1.5	2.4	2.9	3.5
34		0.1	0.3	0.5	0.8	1.1	1.8	2.4	3.0
35			0.2	0.3	0.5	0.8	1.3	1.9	2.5
36			0.1	0.2	0.3	0.6	0.9	1.4	2.0
37				0.1	0.2	0.4	0.6	1.1	1.5
38					0.1	0.3	0.4	0.8	1.1
39						0.2	0.3	0.6	0.8
40						0.1	0.2	0.4	0.5
41							0.1	0.2	0.3
42								0.1	0.2
43									0.1
Total	100.0	100.0	100.0	100.0	100.0	100.0	100.0	100.0	100.0
